# From chelation to transplantation: lessons from a progressive familial intrahepatic cholestasis type 3 case initially managed as Wilson’s disease

**DOI:** 10.1093/gastro/goaf017

**Published:** 2025-01-25

**Authors:** Ali Emre Bardak, Tuğba Kalaycı, Bilger Çavuş, Aslı Çifçibaşı Örmeci, Kadir Demir

**Affiliations:** Department of Internal Medicine, Istanbul Medical Faculty, Istanbul University, Istanbul, Turkey; Department of Internal Medicine, St Elizabeth’s Medical Center, Boston, MA, USA; Department of Medical Genetics, Istanbul Medical Faculty, Istanbul University, Istanbul, Turkey; Division of Gastroenterohepatology, Department of Internal Medicine, Istanbul Medical Faculty, Istanbul University, Istanbul, Turkey; Division of Gastroenterohepatology, Department of Internal Medicine, Istanbul Medical Faculty, Istanbul University, Istanbul, Turkey; Division of Gastroenterohepatology, Department of Internal Medicine, Istanbul Medical Faculty, Istanbul University, Istanbul, Turkey

## Introduction

Progressive familial intrahepatic cholestasis Type 3 (PFIC3) is a rare, autosomal recessive, and hepatocellular-originating cholestatic liver disease caused by mutations in the *ABCB4* gene, which encodes the multidrug resistance protein 3 (MDR3) [1]. The function of the MDR3 protein is to translocate phosphatidylcholine from the inner lipid layer to the outer lipid layer of the bile canaliculus [1]. Phosphatidylcholine combines with bile salts to form mixed micelles, which protect the biliary epithelium from the detergent effects of bile acids. In the absence of MDR3 protein, this translocation is impaired, leaving the biliary epithelium exposed to the detergent effects of bile acids [2]. This disruption leads to cholestasis and progressive liver damage [2].

PFIC3 usually presents in childhood with cholestasis and may progress to cirrhosis [2]. However, adult-onset cases are also seen and can mimic other conditions, like Wilson’s disease (WD) [1, 3]. We reported a 33-year-old male diagnosed with PFIC3, 14 years after being misdiagnosed with WD, the longest reported interval between misdiagnosis and correct diagnosis.

## Case report

A 33-year-old man presented with fatigue, pruritus, and jaundice. He had been diagnosed with WD at age 19 after evaluation for similar symptoms. At that time, liver enzymes were significantly elevated: alanine aminotransferase (ALT) 785 U/L (normal 5–45 U/L), aspartate aminotransferase (AST) 278 U/L (normal 5–42 U/L), gamma-glutamyltransferase (GGT) 366 U/L (normal 5–85 U/L), alkaline phosphatase (ALP) 161 U/L (normal 40–130 U/L), total bilirubin 1.5 mg/dL (normal 0.2–1.2 mg/dL), and conjugated bilirubin 0.7 mg/dL (normal <0.3 mg/dL). International normalized ratio and albumin levels were normal. Serology for viral hepatitis and autoimmune markers, including anti-nuclear antibody, anti-mitochondrial antibody, anti-smooth muscle antibody, anti-neutrophil cytoplasmic antibody, anti-double-stranded DNA, and anti-endomysial antibodies, was negative. Abdominal ultrasound and magnetic resonance cholangiopancreatography revealed a 6-mm gallbladder stone without biliary dilatation. Liver biopsy showed interface hepatitis with periportal inflammation, portal fibrosis, and preserved bile canaliculi. The Knodell histology activity index was 5, and the modified Knodell score was 5. Rhodanine staining indicated hepatic copper accumulation (2,060 μg/g dry weight; normal <55 μg/g). Twenty-four-hour urinary copper excretion was elevated at 211 μg (normal <50 μg/24 h), increasing to 1,497 μg after a penicillamine challenge. Ceruloplasmin was noted as low, but the exact value was not documented. Based on these findings, WD was diagnosed, and penicillamine was initiated.

After 3 years, penicillamine was discontinued due to tremor, and the patient was switched to trientine and zinc. During the 10-year follow-up, liver enzymes fluctuated but remained elevated: ALT 74–1,076 U/L, AST 40–417 U/L, ALP 108–314 U/L, and GGT 97–449 U/L. Conjugated bilirubin ranged from 0.21 to 2.17 mg/dL.

**Figure 1.**
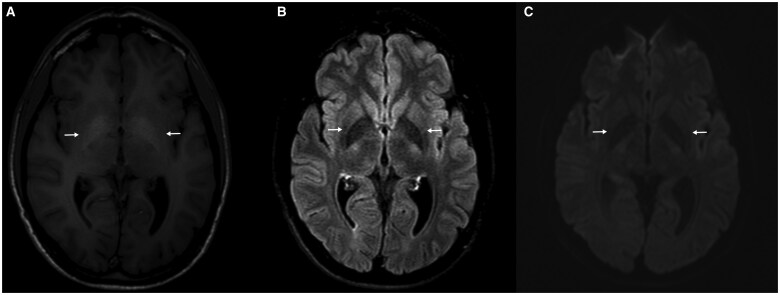
Brain MRI findings. Precontrast T1-weighted images (**A**) demonstrate a symmetric hyperintense signal increase in both globus pallidus (arrow), without a corresponding signal increase on T2-FLAIR (**B**) or diffusion-weighted images (**C**).

At the age of 33, he was referred to our clinic due to inadequate response to the chelation therapy. Laboratory tests showed elevated liver enzymes: ALT 96 U/L, AST 101 U/L, GGT 199 U/L, ALP 260 U/L, total bilirubin 3.92 mg/dL, and conjugated bilirubin 3.81 mg/dL. Autoimmune and viral serology remained negative. Ceruloplasmin was 25 mg/dL (normal 20–60 mg/dL), and serum copper was 134 μg/dL (normal 70–140 μg/dL). Twenty-four-hour urinary copper excretion was 187 μg. Liver biopsy showed cirrhosis, and hepatic copper concentration remained elevated at 1,278 μg/g. Imaging revealed a 15-mm gallbladder stone with normal bile ducts. Ophthalmologic examination found no Kayser–Fleischer rings. Cranial MRI showed basal ganglia changes, which are not typical for WD (Figure 1).

Genetic testing for WD was performed first to confirm its absence, revealing no *ATP7B* mutations. Subsequently, genetic testing for PFIC3, prompted by clinical suspicion, identified compound heterozygous mutations in the *ABCB4* gene, confirming the diagnosis. The patient is currently on the waitlist for a liver transplantation.

## Genetic analysis

Two heterozygous missense variants were detected in the *ABCB4* gene: c.1150G>A (p.Gly384Arg) in exon 11 and c.1529A>G (p.Asn510Ser) in exon 13. Both variants have been previously reported [4, 5]. The segregation analysis confirmed that the patient inherited the c.1150G>A (p.Gly384Arg) variant from the father and the c.1529A>G (p.Asn510Ser) variant from the mother. This pattern of inheritance supports the biallelic nature of *ABCB4* mutations required for the manifestation of PFIC3.

## Discussion

This case highlights the diagnostic challenges when distinguishing WD from PFIC3. Elevated hepatic and urinary copper levels are not exclusive to WD and can occur in cholestatic liver diseases due to impaired biliary copper excretion [1–3].

The absence of Kayser–Fleischer rings, normal ceruloplasmin, and serum copper levels argued against WD, which raised suspicion of a misdiagnosis. The initial diagnosis of WD then was revoked following the lack of *ATP7B* mutations. Genetic testing ultimately confirmed *ABCB4* mutations, establishing the diagnosis of PFIC3. Benign recurrent intrahepatic cholestasis was considered in the differential diagnosis. However, the persistent rather than episodic nature of the patient’s condition made it less likely [6].

It might seem that the patient had a Leipzig Score of 5 at first glance, which is enough (≥4) to establish the WD diagnosis. However, it should be kept in mind that the Leipzig Score should be applied in the absence of cholestasis and hepatitis, which was not the case for our patient [7].

It is important to keep in mind that the significance of cranial imaging findings in WD is still up for debate. The described findings in our case (Figure 1) are considered not typical for WD since they could also potentially be secondary to encephalopathy, accumulation of calcium, or paramagnetic substances such as iron [8]. Tremor is also not a typical feature of PFIC3 and was attributed to penicillamine toxicity in our case [2, 9].

WD and PFIC3 share overlapping features, such as significantly elevated hepatic copper levels and liver dysfunction [3]. In WD, they result from a defect in copper excretion, whereas in PFIC3, they are secondary to cholestasis [1, 7]. Differentiating between these two conditions is crucial because their management strategies differ entirely. Chelation therapy is the mainstay of treatment for WD, while PFIC3 is managed with ursodeoxycholic acid and often requires liver transplantation [1, 2].

It was also shown that the *ABCB4* gene is mutated in more than one-third of the patients who have chronic cholestasis [10]. This finding makes genetic testing for PFIC3 a plausible approach for this patient population.

## Conclusion

PFIC3 should be considered in patients with cholestatic liver disease and copper accumulation, especially when genetic testing for WD is not yet performed or negative. Accurate diagnosis is of utmost importance for timely and appropriate management.

## References

[1] Jacquemin E , De Vree JM, Cresteil D et al The wide spectrum of multidrug resistance 3 deficiency: from neonatal cholestasis to cirrhosis of adulthood. Gastroenterology 2001;120:1448–58. 11313315 10.1053/gast.2001.23984

[2] Davit-Spraul A , Gonzales E, Baussan C et al The spectrum of liver diseases related to ABCB4 gene mutations: pathophysiology and clinical aspects. Semin Liver Dis 2010;30:134–46. 20422496 10.1055/s-0030-1253223

[3] Boga S , Jain D, Schilsky ML. Presentation of progressive familial intrahepatic cholestasis type 3 mimicking Wilson disease: molecular genetic diagnosis and response to treatment. Pediatr Gastroenterol Hepatol Nutr 2015;18:202–8. 26473142 10.5223/pghn.2015.18.3.202 PMC4600706

[4] Degiorgio D , Crosignani A, Colombo C et al ABCB4 mutations in adult patients with cholestatic liver disease: impact and phenotypic expression. J Gastroenterol 2016;51:271–80. 26324191 10.1007/s00535-015-1110-z

[5] Poupon R , Arrive L, Rosmorduc O. The cholangiographic features of severe forms of ABCB4/MDR3 deficiency-associated cholangiopathy in adults. Gastroenterol Clin Biol 2010;34:380–7. 20537830 10.1016/j.gcb.2010.04.011

[6] van der Woerd WL , van Mil SW, Stapelbroek JM et al Familial cholestasis: progressive familial intrahepatic cholestasis, benign recurrent intrahepatic cholestasis and intrahepatic cholestasis of pregnancy. Best Pract Res Clin Gastroenterol 2010;24:541–53. 20955958 10.1016/j.bpg.2010.07.010

[7] European Association for Study of Liver. EASL clinical practice guidelines: Wilson's disease. J Hepatol 2012;56:671–85. 22340672 10.1016/j.jhep.2011.11.007

[8] Rędzia-Ogrodnik B , Członkowska A, Antos A et al Pathognomonic neuroradiological signs in Wilson's disease—truth or myth? Parkinsonism Relat Disord 2023;107:105247. 36543734 10.1016/j.parkreldis.2022.105247

[9] Kumar V , Singh AP, Wheeler N et al Safety profile of D-penicillamine: a comprehensive pharmacovigilance analysis by FDA adverse event reporting system. Expert Opin Drug Saf 2021;20:1443–50. 34259127 10.1080/14740338.2021.1956460

[10] Ziol M , Barbu V, Rosmorduc O et al ABCB4 heterozygous gene mutations associated with fibrosing cholestatic liver disease in adults. Gastroenterology 2008;135:131–41. Published correction appears in *Gastroenterology* 2008;135:1429. 18482588 10.1053/j.gastro.2008.03.044

